# Multikilohertz optical microelastography for viscoelastic characterization of soft biomaterials demonstrated on melanoma spheroids

**DOI:** 10.1117/1.JBO.31.10.106005

**Published:** 2026-10-22

**Authors:** José M Cortés, Inas H Faris, Manuel Hurtado, Jorge Torres, Juan Marchal, Antonio Callejas, Guillermo Rus

**Affiliations:** a University of Granada, Department of Structural Mechanics, Granada, Spain; b Instituto de Investigación Biosanitaria, ibs.GRANADA, Granada, Spain; c University of Granada, Excellence Research Unit “ModelingNature” (MNat), Granada, Spain; d LabTAU, INSERM, Centre Léon Berard, Université Claude Bernard Lyon 1, Lyon, France; e University of Granada, Department of Human Anatomy and Embryology, Granada, Spain; f BioFab i3D Lab - Biofabrication and Three-Dimensional (Bio)printing Laboratory, Granada, Spain

**Keywords:** planar shear-wave microelastography, biomaterial validation, viscoelasticity, kilohertz shear waves, three-dimensional tissue constructs

## Abstract

**Significance:**

The stiffness and viscosity of biological tissue determine how mechanical forces are transmitted and dissipated within it, and both are increasingly used to study tumor growth and treatment response. Measuring these properties within small three-dimensional samples is challenging: it requires shear waves at kilohertz frequencies, which attenuate over short distances, while conventional excitation methods introduce additional signal loss that is difficult to separate from the intrinsic dissipation of the tissue.

**Aim:**

To enable sub-millimeter viscoelastic characterization of three-dimensional biological constructs in the multikilohertz frequency range using quasi-planar wave excitation and optical detection.

**Approach:**

We developed an optical microelastography platform that combines quasi-planar shear-wave excitation using a piezoelectric actuator, high-speed imaging (up to 210,000 fps), and k-space dispersion analysis to estimate frequency-dependent viscoelastic parameters through a Kelvin–Voigt model. The system was validated using polyacrylamide phantoms with tunable viscoelastic properties at five concentrations (15% to 26% v/v).

**Results:**

Validation across polyacrylamide concentrations yielded effective shear moduli of 0.70 to 19.31 kPa, with a minimum resolvable shear wavelength of ∼110  μm in the polyacrylamide phantoms. Experimental phase-velocity dispersion showed good agreement with finite-element predictions (RMSE<0.16  m/s, mean relative error <3%, $R^{2}>0.96$). Measurements on four melanoma spheroids (237 to 823 µm diameter) produced effective viscoelastic parameters of μ=0.45 to 0.71 kPa and η=0.033 to 0.097  Pa·s ($R^{2}\geq 0.96$), obtained within specimen-specific analysis bands and consistent with values reported for three-dimensional tumor spheroid models.

**Conclusions:**

This platform demonstrates kilohertz-range viscoelastic characterization of three-dimensional biological constructs at sub-millimeter scales, validated in polyacrylamide phantoms and demonstrated in proof-of-concept measurements on four melanoma tumor spheroids of different sizes. The quasi-planar excitation approach reduces geometric spreading artifacts associated with conventional excitation methods and provides a noninvasive optical tool for studying frequency-dependent mechanical behavior in mechanobiology and tissue engineering.

## 1 Introduction

Viscoelastic properties of cellular environments are increasingly recognized as important contributors to tumor growth, invasion, and treatment response.^1^^–^^4^ The mechanical microenvironment regulates cell fate, tissue function, and disease progression through mechanotransduction processes.^5^ Malignant tissues typically exhibit increased bulk stiffness compared with healthy tissues,^6^ whereas individual cancer cells can be more compliant than their normal counterparts.^7^ It is at multicellular length scales (tens to hundreds of micrometres) that mechanical properties relate directly to collective cellular behavior and tissue organization.^8^^,^^9^

To characterize microscale viscoelastic properties, shear-wave elastography provides a non-invasive approach in which wave speed reflects the elastic response, whilst wave attenuation reflects viscous dissipation. Achieving wavelengths comparable to multicellular structures typically requires multikilohertz frequencies. Broadband signals are particularly desirable, as soft tissues exhibit strong frequency-dependent behavior that is best characterized through dispersion analysis.^10^ However, two fundamental constraints limit current implementations.

First, shear-wave attenuation in viscoelastic media increases strongly with frequency, limiting propagation distance and reducing the signal-to-noise ratio, which complicates quantitative dispersion analysis and viscous loss estimation.^11^

Second, conventional excitation methods, such as point or line sources, introduce geometric spreading, an amplitude decay inversely proportional to propagation distance due to energy conservation. This effect superimposes geometric attenuation on intrinsic material dissipation, thereby confounding estimates of viscous losses.

Together, these two factors have historically limited quantitative elastography in the near-ultrasonic frequency regime.

Existing techniques illustrate these limitations in spatial scale and excitation frequency. Magnetic resonance elastography (MRE) operates at frequencies typically below 500 Hz,^12^ producing wavelengths that are too large (millimetres) for microscale interrogation. Conventional ultrasound-based shear-wave elastography (SWE) operates at higher frequencies but provides lateral resolution governed by the acoustic beam width (hundreds of micrometres).^6^ Variants employing continuous excitation, such as harmonic motion imaging,^13^^,^^14^ continuous SWE,^15^^,^^16^ and vibrational SWE,^17^ extend measurement capabilities but remain constrained by similar spatial-resolution and attenuation limitations.

At the opposite extreme, atomic force microscopy (AFM) achieves nanoscale resolution^18^ but is inherently a point-by-point surface-based technique that primarily probes quasi-static mechanical responses. Brillouin microscopy achieves subcellular resolution through noncontact light scattering^19^ operating at gigahertz frequencies, but does not directly provide bulk viscoelastic parameters such as the shear modulus.

Recent advances in optical elastography have expanded measurement capabilities. Optical coherence elastography (OCE) provides extremely high temporal sampling, with detector acquisition rates reaching the megahertz range.^20^ However, it is important to distinguish between detector acquisition rate and mechanical excitation frequency. Although detection speeds can be very high, the mechanical excitation frequencies used in most OCE implementations remain restricted to sub-kilohertz and low-kilohertz ranges (typically 0.1 to 5 kHz).^21^

Recent surface-wave implementations have extended excitation frequencies to ∼10  kHz.^22^ However, like other OCE studies, they rely on surface-confined wave propagation rather than bulk shear-wave propagation through volumetric constructs.^23^

Consequently, despite substantial advances in optical detection sensitivity,^24^^,^^25^ achieving the combination of bulk shear-wave propagation, multikilohertz excitation, and sub-millimeter spatial resolution required for microscale tissue characterization remains challenging.

Tumor spheroids represent physiologically relevant three-dimensional models of solid tumor biology^26^ and provide a well-suited system for microscale viscoelastic characterization, given their known mechanical heterogeneity^27^ and the established association of mechanical cues with phenotypic transitions and treatment resistance.^28^^,^^29^

Their mechanical properties have been characterized with contact methods such as atomic force microscopy^18^ and micropipette aspiration,^30^ and, more recently, with quasi-static compression-based optical coherence elastography—quantitative micro-elastography and its high-resolution variant—which has mapped the three-dimensional elasticity of tumor spheroids.^31^^,^^32^ These optical methods recover an effective elastic modulus from the quasi-static strain response. Most closely related to the present work, optical micro-elastography has recovered the elasticity of single living cells from high-speed tracking of mechanically induced shear waves,^33^ combining a localized source with noise-correlation velocity estimation at the single-cell scale; here, instead, we use quasi-planar propagating shear waves and frequency–wavenumber analysis to recover Kelvin–Voigt parameters (μ, η) at the scale of whole tumor spheroids.

At this scale we need to deal with certain frequency bandwidth constraints. At low frequency, the target geometry sets the lower bound of the usable band. To resolve the shear mechanics of the spheroid rather than those of the surrounding medium, the shear wavelength $\lambda_{s}=c_{s}/f$ should be comparable to or smaller than the spheroid diameter D, typically a few hundred micrometers (i.e., $f≳c_{s}/D$); a wavelength much larger than the spheroid leaves it effectively sub-wavelength, limiting how well its mechanics can be resolved.^34^ The upper bound, in turn, is set by the frequency-dependent attenuation described above, which limits propagation distance and the usable bandwidth within the construct.

In addition, the structural heterogeneity of these constructs poses a further difficulty: at kilohertz frequencies, separating intrinsic material dissipation from geometric artifacts in such highly attenuating and geometrically complex microenvironments remains difficult.^35^

To address these challenges, we modify and extend our previously reported optical microelastography platform, which employed magnetic excitation,^36^ by implementing a piezoelectric quasi-planar shear-wave excitation configuration. The proposed system combines broadband chirp excitation (5 to 25 kHz), high-speed imaging (210,000 fps), and k-space dispersion analysis to estimate frequency-dependent viscoelastic parameters.

The platform is first validated using polyacrylamide phantoms spanning a wide stiffness range (0.70 to 19.31 kPa) and subsequently applied to melanoma tumor spheroids of different sizes as a proof-of-concept demonstration in a biologically relevant construct.

## 2 Materials and Methods

### 2.1 Optical Microelastography Platform

The excitation signal generated by the actuator was characterized with laser Doppler vibrometry (OFV series; Polytec GmbH, Waldbronn, Germany), using a chirp excitation from 15 to 30 kHz (Fig. 1). Peak actuator displacements reached 6.9  μm.

**Fig. 1:**
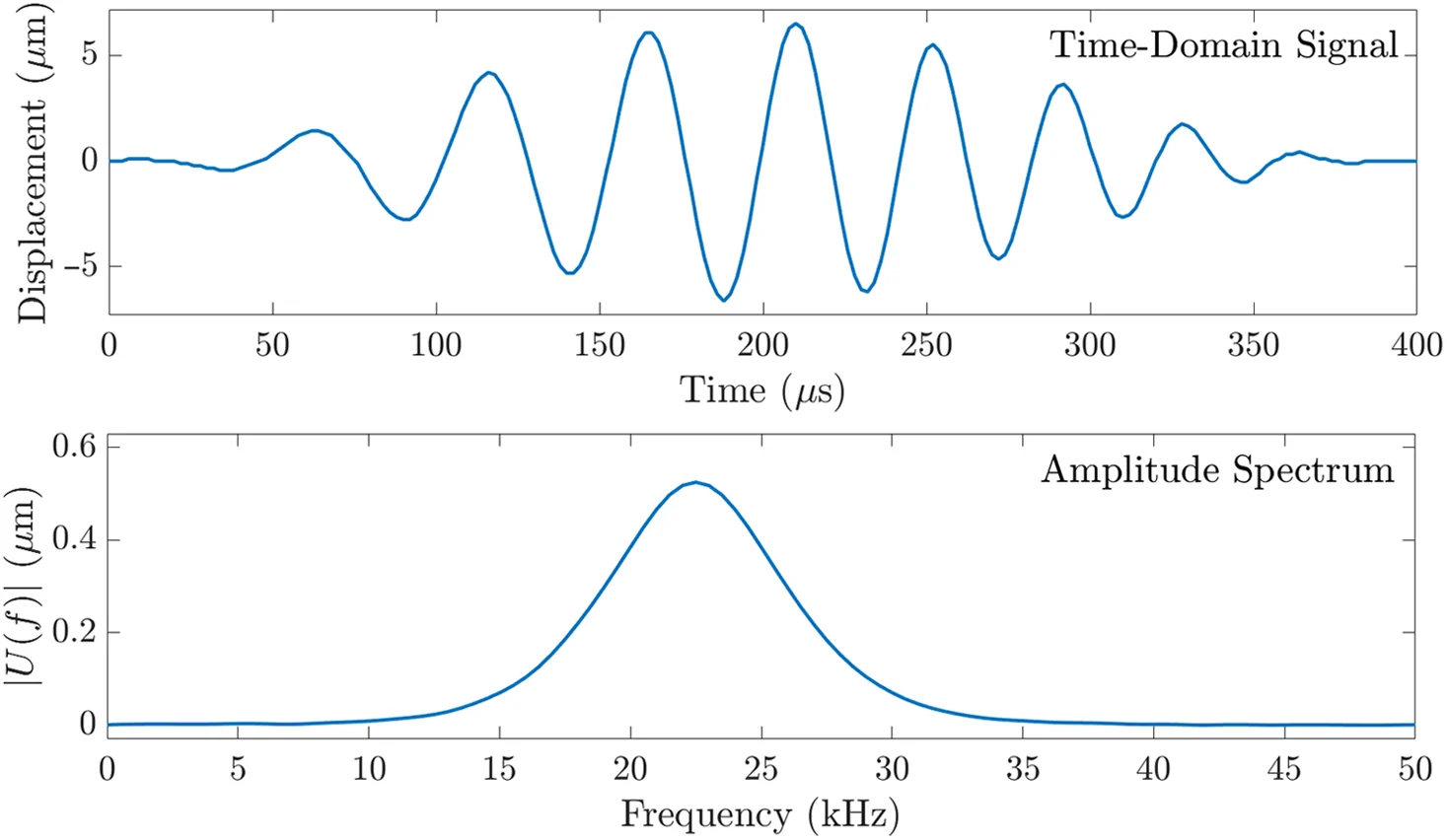
Characterization of the excitation signal generated by the piezoelectric actuator (Hann-windowed chirp, 15 to 30 kHz). Top: time-domain signal measured by the laser Doppler vibrometer and recorded with the oscilloscope. Bottom: Fourier transform of the signal, showing frequency content that closely matches the input chirp excitation.

The experimental platform comprised an excitation subsystem for generating shear waves and an imaging subsystem for recording shear-wave propagation via high-speed optical imaging, both controlled by a PC (Fig. 2).

**Fig. 2:**
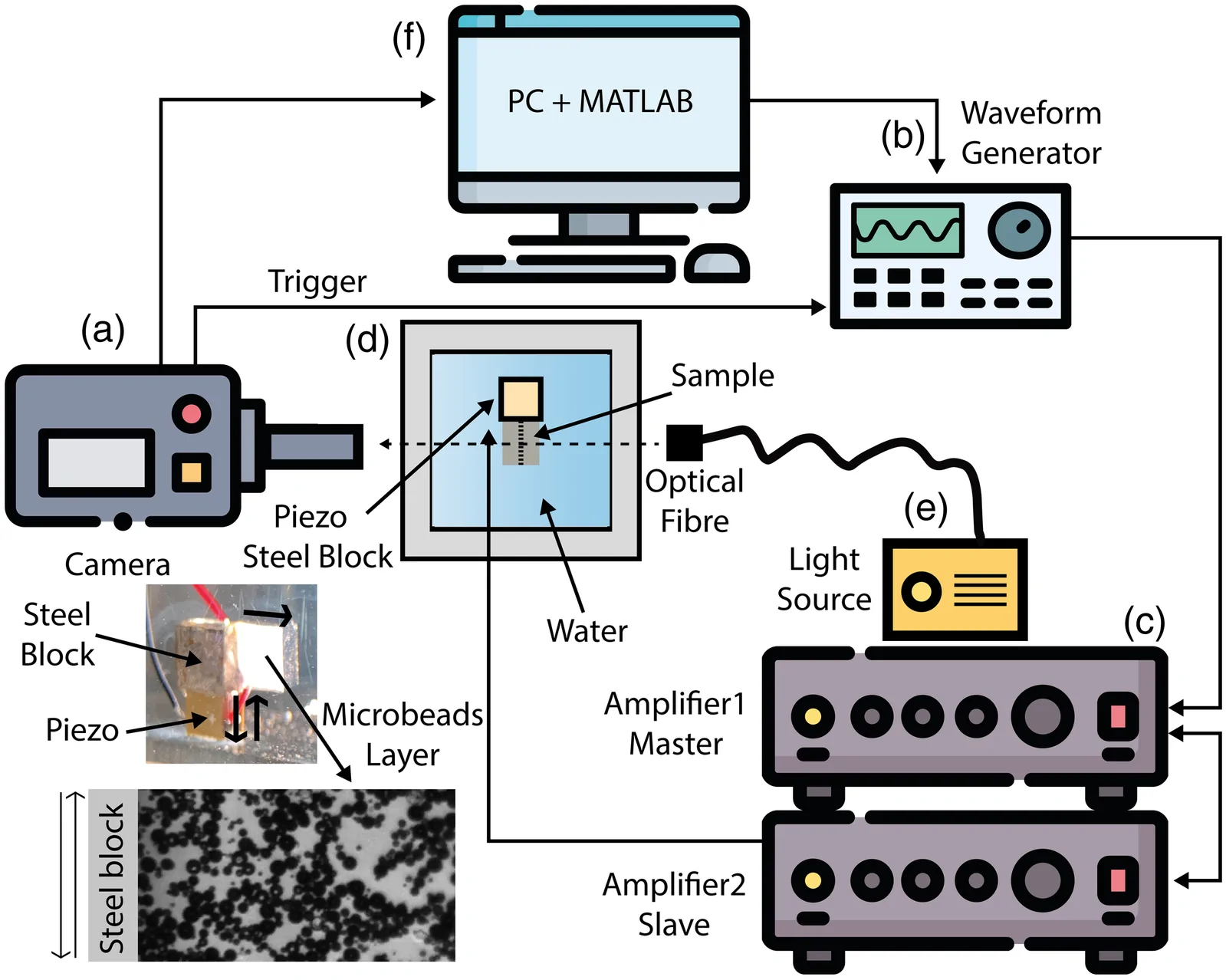
Schematic of the optical microelastography platform. (a) A high-speed camera triggers the function generator while recording image sequences of the sample. (b) The generator outputs a broadband chirp excitation. (c) Two power amplifiers connected in series increase the signal to 150 V peak-to-peak. (d) A piezoelectric actuator immersed in distilled water and capped with a stainless-steel block converts the electrical signal into vertical displacement. (e) A halogen light source coupled to an optical fiber bundle provides collimated illumination. (f) Recorded image sequences are processed in Matlab to extract sample displacements.

Excitation signals were generated using a function generator (33500B Series; Keysight Technologies, Santa Rosa, California, United States) and amplified to 150 V peak-to-peak using two power amplifiers (Model 7224; AE Techron Inc., Elkhart, Indiana, United States) connected in series to accommodate the capacitive load of the piezoelectric actuator.

Shear-wave propagation was recorded at up to 210,000 fps using a high-speed camera (FASTCAM SA-Z; Photron Ltd., Tokyo, Japan) equipped with interchangeable zoom lenses depending on the required field of view. Transillumination was provided by a halogen light source (OSL2; Thorlabs Inc., Newton, New Jersey, United States) coupled to a collimated fiber-optic bundle [Fig. 2(e)].

The piezoelectric actuator was mounted at the bottom of a custom tank with optically transparent side walls, filled with distilled water to provide an optical path for transillumination. A stainless-steel cube was rigidly attached to the top surface of the actuator, and the sample was fixed to one side of the cube. This configuration efficiently transmitted the actuator’s vertical motion to the sample, generating shear waves that propagated horizontally [Fig. 2(d)].

Water immersion minimized optical distortion and refraction artifacts.^24^^,^^25^ The high-speed camera served as the master trigger, initiating both image acquisition and waveform emission simultaneously, thereby ensuring temporal alignment between the first recorded frame and the onset of wave excitation [Figs. 2(a) and 2(b)].

### 2.2 Experimental Protocols

#### 2.2.1 Sample preparation

To validate the system using materials with tunable viscoelastic properties, polyacrylamide hydrogels were prepared at concentrations of 15%, 17%, 20%, 23%, and 26% (v/v). Polymerization was initiated with ammonium persulfate (APS; Sigma-Aldrich, St. Louis, Missouri, United States) and catalyzed by N,N,N’,N’-tetramethylethylenediamine (TEMED; Sigma-Aldrich, St. Louis, Missouri, United States) in an acrylamide/bis-acrylamide solution (19:1 ratio, 40% w/v; Omnipur A9926, Merck Millipore, Darmstadt, Germany), following an optimized formulation from previous work.^36^

The APS and TEMED contents were adjusted to 0.84% (w/v) and 0.5% (v/v), respectively, to improve gel consistency. To enable displacement tracking, hollow borosilicate glass microspheres (diameter≤25  μm; Product Code S-HGB-015, LordsWorld Srls, Desenzano del Garda, Brescia, Italy) were embedded within a thin central layer to provide brightfield transmission.

For the proof-of-concept biological application, melanoma spheroids were cultured from the MEL-1 cell line (BBSPA-Mel#1, Biobank of the Andalusian Public Health System, Spain) following standard ultra-low-attachment protocols. Cells were pelleted by centrifugation, the supernatant was removed, and the cells were resuspended in fresh culture medium. Following trypan blue viability assessment (0.4%), ∼1000 viable cells were seeded into each well of round-bottom ultra-low-attachment 96-well plates (maximum volume 200  μL). A partial medium exchange (100  μL) was performed 24 h after seeding, and spheroid assembly was typically completed within 48 h.^37^ Spheroids of different sizes were obtained by varying the seeding density (≈1000 to 20,000 viable cells per well): the ≈1000-cell protocol described above yielded the smaller spheroids (a, b; 237 to 277  μm), while higher seeding densities produced the larger spheroids (c, d; 660 to 823  μm).

The fully formed spheroids were embedded in a 23% polyacrylamide gel to enable shear-wave propagation (Fig. 3). This gel concentration was selected to balance mechanical support with efficient wave transmission. Because experimental constraints prevented positioning the spheroid close to the excitation region, the 23% formulation provided sufficiently low attenuation to ensure adequate shear-wave energy at the measurement location.

**Fig. 3:**
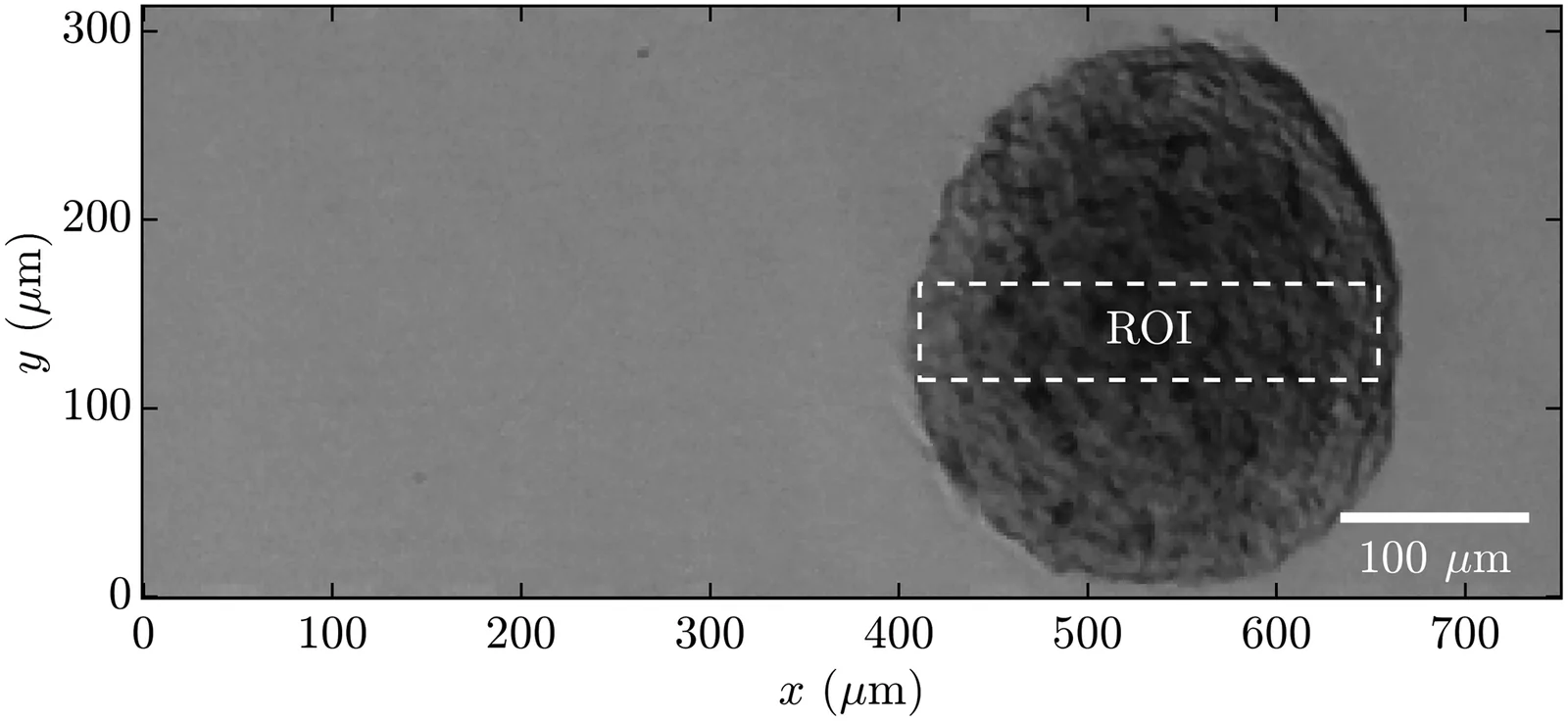
Tumor spheroid with a diameter of 277  μm embedded in 23% polyacrylamide gel. The spheroid is located ∼400  μm from the stainless-steel coupling block used for excitation (position x=0). Spatial coordinates are referenced using the 100  μm scale bar.

The region of interest (ROI) was defined within the central equatorial band of each spheroid, where local curvature is minimal and wavefronts can be approximated as locally planar.

All measurements were performed at ambient temperature (22 to 24°C); no active thermal control was applied.

#### 2.2.2 Data acquisition protocol

Different excitation bandwidths were employed for phantoms and spheroids to optimize the trade-off between spectral coverage and signal-to-noise ratio. For phantom validation, three chirp signals were applied (5 to 15 kHz, 10 to 25 kHz, and 15 to 30 kHz) to assess viscoelastic behavior across a broad frequency range.

For spheroid characterization, the excitation was adapted to specimen size, following the wavelength criterion described in the introduction. For the smaller spheroids (a, b; 237 to 277  μm), a 10 to 25 kHz chirp was used, ensuring that the shear wavelengths remained smaller than the spheroid diameter while avoiding excessive attenuation. For the larger spheroids (c, d; 660 to 823  μm), whose size admits longer wavelengths, a single-cycle 5 kHz sinusoidal pulse was used; its short duration (0.2 ms) produces a broadband spectrum, of which the 2 to 6 kHz range, where the dispersion ridge is well defined, was used for the analysis.

The chirp excitations were windowed using a Hann function to reduce spectral leakage and provide gradual amplitude transitions, thereby limiting transient artifacts at the start and end of the excitation. In contrast, the single-cycle 5 kHz pulse used for the larger spheroids was applied without additional windowing.

Recordings were acquired at 210,000 fps for the phantom and small-spheroid measurements, and at 100,000 fps for the large-spheroid measurements; both satisfy the Nyquist–Shannon sampling criterion for the analyzed frequencies. Magnification and field of view (FOV) were adjusted to each measurement. A 5× objective was used for the phantom and small-spheroid measurements (FOV≈750  μm) and for spheroid (c) (FOV≈1240  μm), and a 3× objective for the larger spheroid (d) (FOV≈2050  μm).

The object-space pixel pitch was ≈1.95  μm for the phantom and small-spheroid sequences (5×, 210,000 fps), ≈1.95  μm for spheroid (c) (5× with a wider field of view at 100,000 fps, set with the zoom lens), and ≈3.25  μm for spheroid (d) (3×, 100,000 fps).

### 2.3 Data Processing and Viscoelastic Parameter Inversion

The high-speed image sequences were processed using custom scripts in Matlab (MathWorks, Inc.) following a multistep pipeline that converts recorded image sequences into quantitative viscoelastic parameters. The overall workflow is illustrated in Fig. 4.

**Fig. 4:**
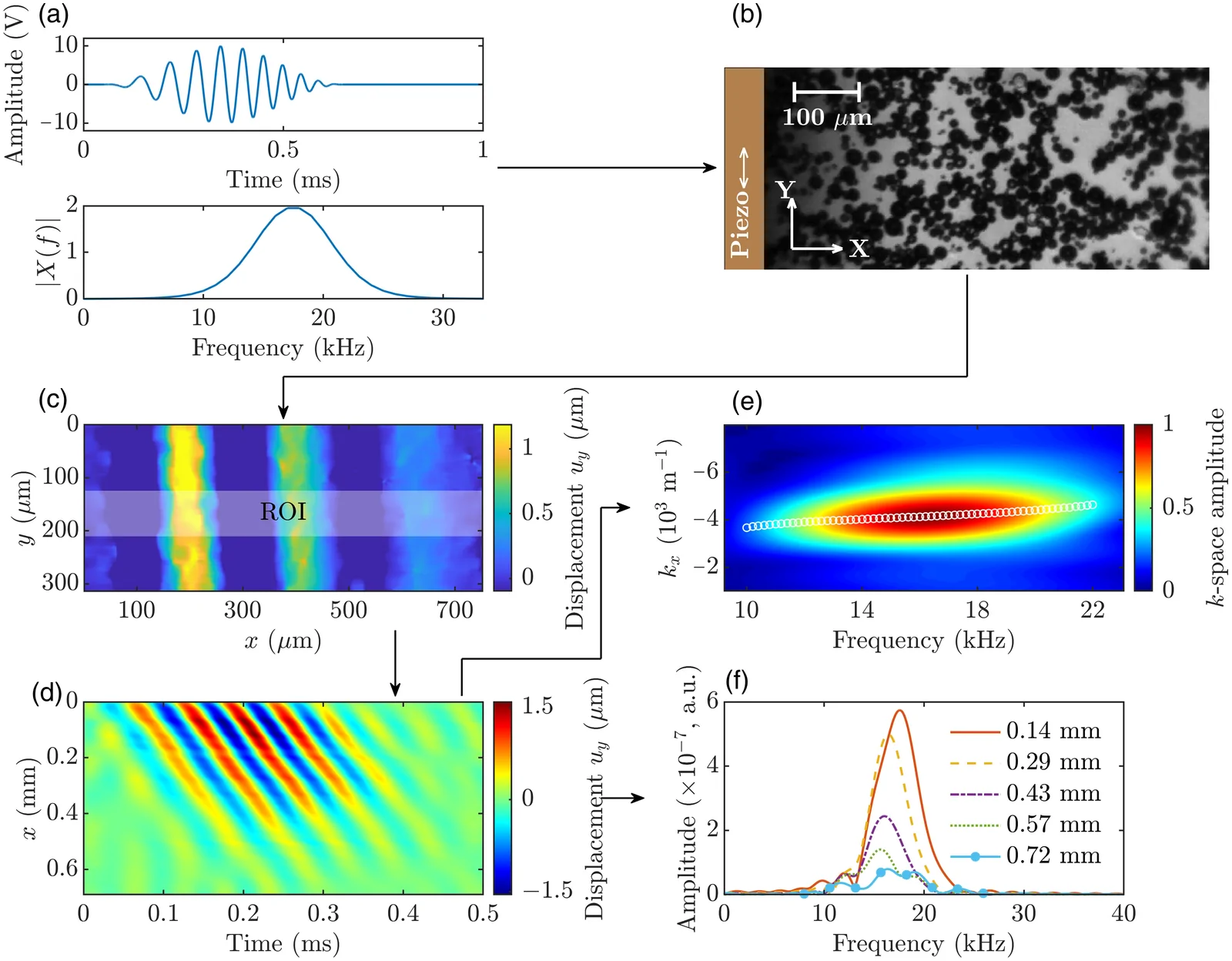
Processing workflow for high-frequency plane shear-wave measurements in a 23% polyacrylamide sample. (a) Broadband chirp excitation tapered with a Hann window (10 to 25 kHz). (b) The excitation is transmitted through a piezoelectric actuator to the polyacrylamide gel containing a thin interlayer of borosilicate microbeads (diameter≤25  μm) used for brightfield transmission. (c) Two-dimensional displacements are estimated using a dense optical flow algorithm, with shear-wave propagation along the x-direction and particle motion primarily along the y-direction. (d) Region of interest covering the full field of view, visualized in a spatiotemporal displacement map. (e) The displacement field is transformed into k-space using a two-dimensional Fourier transform, from which the dispersion curve is extracted. (f) Spectral comparison showing close agreement between the generated and recorded excitation signals.

The frame-to-frame vertical displacement field $u_{y}(x,y,t)$ was estimated using the dense Farnebäck optical flow method.^38^ This approach was selected because it provides a spatially continuous displacement field with sufficient resolution to capture high-frequency shear waves while minimizing spatial aliasing.

The algorithm was implemented with parameters tuned to balance noise suppression and phase preservation (pyramidal scaling 0.5, three pyramid levels, 15-pixel window, three iterations, and polynomial expansion with n=5 and σ=1.2). This configuration produced spatially continuous displacement fields while preserving the phase information.

The 15-pixel optical-flow window set an effective spatial resolution of  $\approx 30\times 30\;\mu m^{2}$ for displacement analysis at 5×, scaling proportionally at 3×. Across phantoms and spheroids, the shear wavelengths analyzed (≈114 to 385  μm) correspond to angular wavenumbers $k_{x}=2\pi /\lambda \approx 1.6$ to 5.5×104  rad/m, far below the spatial Nyquist limits ($\pi /\Delta x\approx 1.6\times 10^{6}\;\mathrm{rad}/m$ at 5× and $\approx 9.8\times 10^{5}\;\mathrm{rad}/m$ at 3×).

The ROI was selected so that its spatial extent L set a wavenumber resolution $\Delta k_{x}\approx 2\pi /L$ fine enough to localize the dispersion ridge $k_{x}(f)$ over the analyzed frequency band, while averaging over the transverse (y) direction, orthogonal to propagation, improved the signal-to-noise ratio and reduced measurement uncertainty.

To isolate forward-propagating waves, a directional filter in the 2D frequency–wavenumber domain removed counter-propagating reflections by setting the corresponding wavenumber components to zero.^39^ A Hann window was applied to the edges of the directional mask to avoid sharp cuts of frequency components.

The directionally filtered field $u_{y}(x,t)$ was transformed into the frequency–wavenumber domain $\left(k_{x},\omega \right)$ using a two-dimensional FFT to extract the dispersion relation.^40^ Shear-wave energy formed a spectral ridge in this domain, and for each angular frequency ω, the peak wavenumber $k_{x}(\omega )$ was identified. From this, the phase-velocity dispersion curve $c_{s}(\omega )$ was then obtained from the standard relation $c_{s}(\omega )=\omega /k_{x}(\omega )$. The minimum resolvable wavelength $\lambda_{\min}$ was estimated from the maximum resolved wavenumber in the dispersion map.

For viscoelastic characterization, the classical Kelvin–Voigt (KV) model (μ,η) was adopted because it provides two interpretable parameters and is widely used for biomaterial characterization.^10^

To evaluate whether a more general rheological description was required, the dispersion curves were also fitted using the fractional Kelvin–Voigt model with exponent (0≤α≤1). The estimated exponents remained close to unity (α=0.81 to 1.09), indicating that the measured behavior is well approximated by the classical Kelvin–Voigt formulation. Although soft biological materials often exhibit apparent power-law rheology, fractional models reduce locally to their integer-order limit over sufficiently narrow frequency ranges.

In our measurements, the accessible frequency band spans ∼0.70 decades (5 to 25 kHz), within which the fractional dispersion becomes practically indistinguishable from Kelvin–Voigt behavior. Previous studies have similarly reported reliable Kelvin–Voigt performance in the kilohertz regime relevant to optical elastography measurements.^23^^,^^41^^,^^42^

The spatiotemporal representation in Fig. 4(d) shows frequency-dependent attenuation with propagation distance, with higher-frequency components decaying more rapidly than lower-frequency components. This behavior is consistent with viscous losses predicted by the Kelvin–Voigt model, for which the attenuation coefficient scales as $\alpha (\omega )\propto \omega^{2}\eta /(2\mu c_{s})$. These observations indicate that the measurements capture attenuation trends expected from viscoelastic dissipation.

### 2.4 Numerical Verification and Analysis Framework

To verify the reliability and internal consistency of the data-processing and inversion pipeline, a finite element model was implemented in COMSOL Multiphysics^®^ v6.2. Synthetic displacement fields were generated for viscoelastic media with parameters representative of the experimentally observed range under controlled, noise-free conditions, and were analyzed to assess the retrieval accuracy of μ and η.

Perfectly matched layers were placed along the top, bottom, and right boundaries to suppress reflections, and a prescribed vertical displacement of 10  μm was applied at the left boundary. Single-frequency harmonic excitations spanning 5 to 50 kHz (for testing upper limits) in 5 kHz steps were imposed, emulating individual spectral components of the experimental excitation. For each frequency, a steady-state frequency-domain analysis computed the harmonic displacement field to characterize attenuation and dispersion.

The finite-element mesh used rectangular quadratic Lagrange elements, which provide higher accuracy than linear elements. A density of 10 elements per minimum wavelength ($\lambda_{\min}$) ensured accuracy at the highest simulated frequency. A direct frequency-domain solver (MUMPS) computed the steady-state response.

Shear-wave velocities were obtained directly from the COMSOL results using a phase-gradient approach.^43^ The complex displacement fields u(x,ω) were extracted via LiveLink, and a region of interest comparable to the experimental field of view was selected (Fig. 5).

**Fig. 5:**
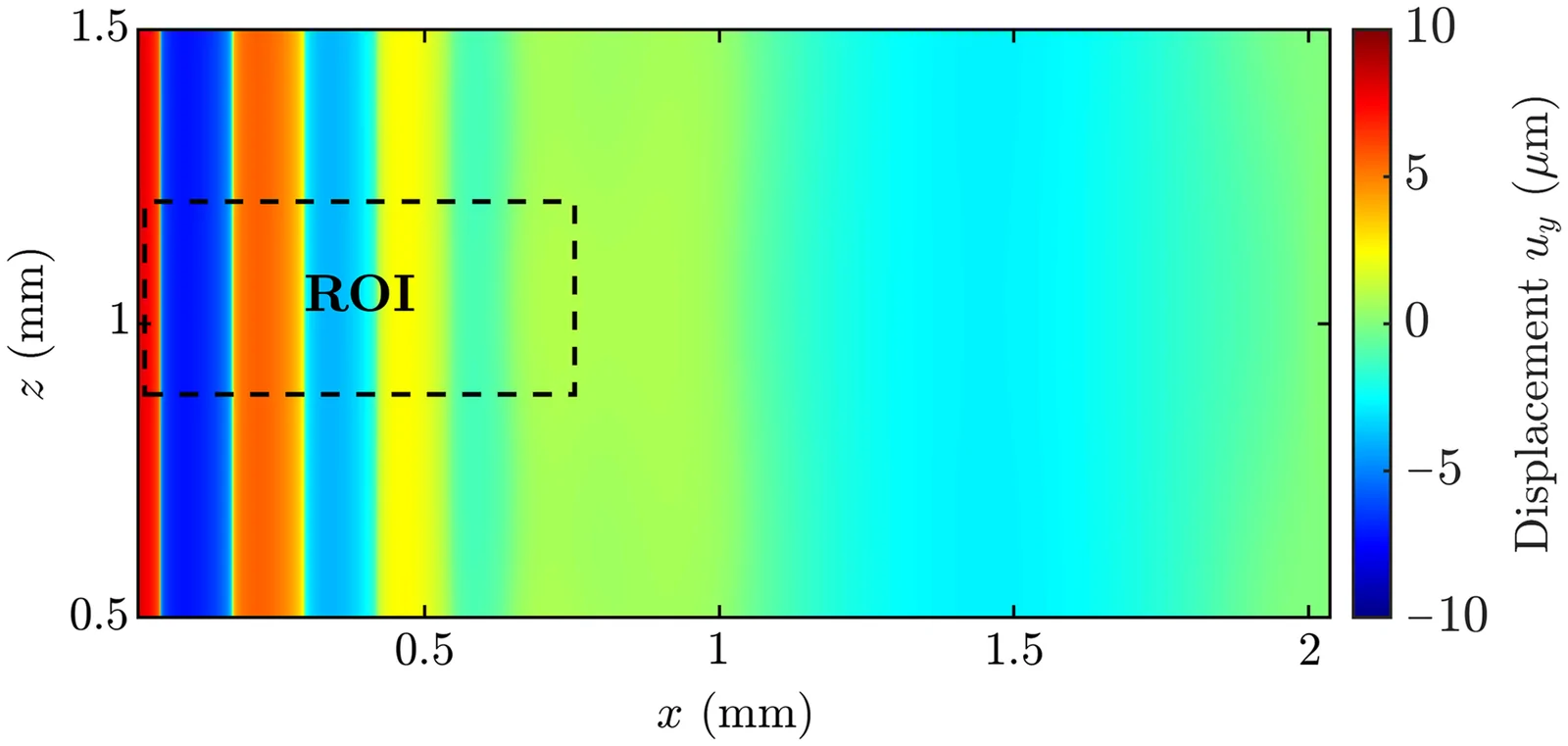
Simulated shear-wave displacement field ($u_{y}$) in a 23% polyacrylamide gel model obtained from the frequency-domain finite element simulation. The dashed box indicates the region of interest (ROI), selected to match the typical experimental field of view.

This phase-gradient method provides an independent verification of the COMSOL frequency-domain results. In contrast, the experimental time-domain displacement data were processed using the k-space framework described in Sec. 2.3. Agreement between the velocities obtained from both methods and the prescribed material parameters supports the accuracy of the experimental measurements and the overall inversion framework (see Results, Tables 1 and 2).

**Table 1:** Effective viscoelastic parameters obtained from model fitting across 5 to 25 kHz: shear modulus (μ), viscosity (η), minimum resolvable wavelength ($\lambda_{\min}$), and fractional exponent (α). Values of α close to unity support the use of the classical Kelvin–Voigt approximation within the studied bandwidth.

Conc. (%)	μ (kPa)	η (Pa·s)	$\lambda_{\min}$ (μm)	$R^{2}$	${\mathrm{CV}}_{\mu }$ (%)	${\mathrm{CV}}_{\eta }$ (%)	KV_frac α
15	0.70±0.24	0.027±0.002	113.96	0.97 to 0.98	34.5	7.1	1.093±0.053
17	1.94±0.37	0.060±0.007	164.64	0.95 to 0.97	19.1	12.4	0.973±0.047
20	4.67±0.48	0.103±0.001	202.36	0.92 to 0.96	10.3	1.2	0.931±0.063
23	10.14±0.72	0.145±0.029	232.70	0.95 to 0.96	7.1	20.3	0.809±0.048
26	19.31±2.71	0.215±0.023	277.81	0.88 to 0.98	14.1	10.7	0.852±0.151

**Table 2:** Simulation values of elasticity (μ) and viscosity (η) at different concentrations according to the Kelvin–Voigt model, the minimum wavelength achieved ($\lambda_{\min}$), and quantitative FEM–experimental agreement metrics.

Concentration (%)	μ (kPa)	η (Pa·s)	$\lambda_{\min}$ (μm)	RMSE (m/s)	Error (%)	$R^{2}$
15	0.70	0.027	108.38	0.063	2.14	0.983
17	1.92	0.062	162.66	0.069	1.95	0.989
20	4.68	0.102	204.37	0.101	2.24	0.983
23	10.15	0.144	232.42	0.133	2.53	0.968
26	19.25	0.213	286.98	0.152	2.47	0.962

### 2.5 Data Analysis

All quantitative data are presented as mean ± standard deviation (SD). For phantom validation, three independently prepared samples were analyzed for each hydrogel concentration. Each phantom was polymerized as a separate batch, and measurements were performed on comparable central regions of interest.

Measurements were performed on four melanoma spheroids of the MEL-1 cell line, cultured in independent batches and spanning a range of sizes (≈1000 to 20,000 seeded cells; 237 to 823  μm in diameter), as a proof-of-concept demonstration of technical feasibility. The number of specimens is limited by the requirement to position each spheroid within the shear-wave propagation range at kilohertz frequencies, which constrains the throughput of the measurement.

No statistical hypothesis testing was performed, as the primary objective of this study is methodological validation of the measurement platform rather than biological inference.

## 3 Results

### 3.1 Viscoelastic Properties of Phantoms

The effective shear modulus (μ) and viscosity (η) obtained by fitting the Kelvin–Voigt model to the experimental dispersion data across 5 to 25 kHz are summarized in Table 1.

Both parameters increased monotonically with polyacrylamide concentration. For example, the shear modulus increased from 0.70±0.24  kPa for the 15% gel to 19.31±2.71  kPa for the 26% gel. Similarly, the viscosity increased from 0.027±0.002 to 0.215±0.023 Pa·s across the same concentration range.

Kelvin–Voigt model fits showed good agreement across all concentrations ($R^{2}>0.88$, Table 1). For the shear modulus, the coefficient of variation ($CV_{\mu }$) generally decreased with increasing concentration (from 34.5% to 14.1%), indicating improved measurement reproducibility at higher gel stiffness. In contrast, $CV_{\eta }$ exhibited greater variability, ranging from 1.2% to 20.3%, but remained within acceptable limits for viscoelastic characterization.

The frequency dependence of the phase velocity ($c_{s}$) and wavelength (λ) across concentrations is shown in Fig. 6(a). Phase velocity increased monotonically with frequency for all concentrations. At any given frequency, higher polyacrylamide concentrations yielded higher phase velocities, consistent with the increasing stiffness values reported in Table 1.

**Fig. 6:**
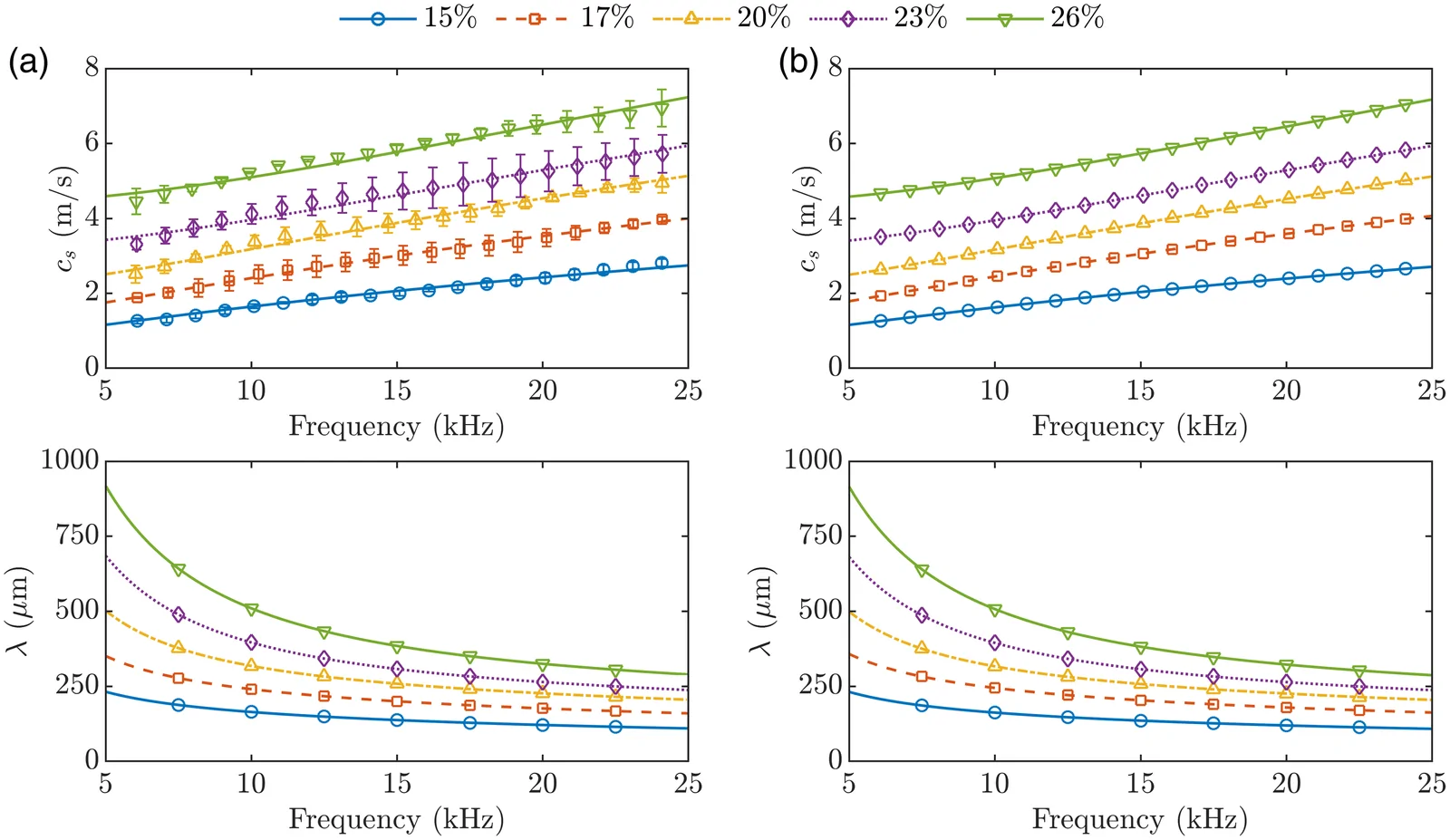
Comparison of shear-wave phase velocity ($c_{s}$, top) and wavelength (λ, bottom) as functions of frequency (5 to 25 kHz) in polyacrylamide gels with concentrations ranging from 15% to 26%. (a) Experimental results obtained from polyacrylamide phantoms, with wavelengths calculated from the measured wavenumbers. (b) Finite element simulations performed in COMSOL. Both datasets show increasing $c_{s}$ with frequency and gel concentration, with a corresponding decrease in λ.

The minimum resolvable shear wavelengths ($\lambda_{\min}$) (Table 1) ranged from 113.96 to 277.81  μm across phantom concentrations. This implies that, theoretically, if spheroids showcase properties on the same range, it will be possible to perform mechanical interrogation at multicellular length scales.

Error bars represent the standard deviation across N=3 independent replicates.

### 3.2 Numerical Simulation Verification

The simulations reproduced the main features observed experimentally, including the frequency-dependent behavior of shear-wave propagation (Fig. 6).

The phase-velocity dispersion curves ($c_{s}(\omega )$) obtained from the simulation data [Fig. 6(b)] exhibited trends with frequency and concentration consistent with the experimental results [Fig. 6(a)].

Quantitative comparison between experimental and FEM phase-velocity dispersion curves showed strong agreement across all concentrations, with root mean square errors below 0.16  m/s (mean relative error <3%) and coefficients of determination $R^{2}>0.96$ (Table 2). These results are consistent with the k-space inversion methodology and the Kelvin–Voigt constitutive model within the studied frequency range.

Analysis of the simulated amplitude decay (Fig. 7) showed that high-frequency shear waves attenuate more rapidly and that waves propagate further in stiffer gels (e.g., 23% concentration) than in softer gels (e.g., 15% concentration), which is consistent with theoretical predictions.

**Fig. 7:**
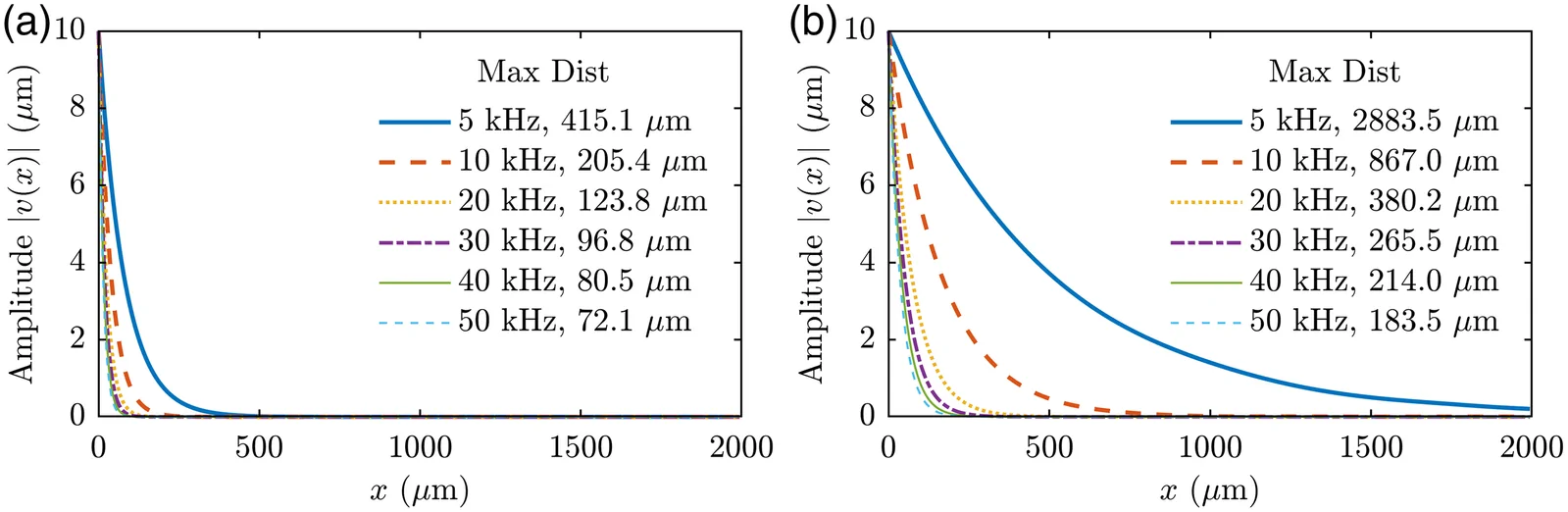
Comparison of simulated shear-wave amplitude decay in polyacrylamide gels obtained from COMSOL simulations. Panel (a) shows results for a 15% gel, whereas panel (b) shows results for a 23% gel across excitation frequencies of 5 to 50 kHz. Curves are distinguished by line style and line width in addition to color. Max Dist is the propagation distance at which the amplitude falls to 0.05  μm. Greater attenuation (faster amplitude decay) is observed in the softer 15% gel compared with the stiffer 23% gel.

### 3.3 Shear Wave Propagation and Mechanics of Tumor Spheroids

The microelastography system was then applied to demonstrate feasibility in melanoma tumor spheroids of different sizes (237 to 823  μm diameter) embedded in 23% polyacrylamide gel, as described in Sec. 2.2.

The system captured shear-wave propagation through the spheroid structure, with the cellular architecture providing sufficient endogenous contrast for the optical flow algorithm to track displacements without the need for exogenous contrast agents. Figure 8 shows representative snapshots of the spatiotemporal displacement field ($u_{y}$) during shear-wave propagation through a representative spheroid.

**Fig. 8:**
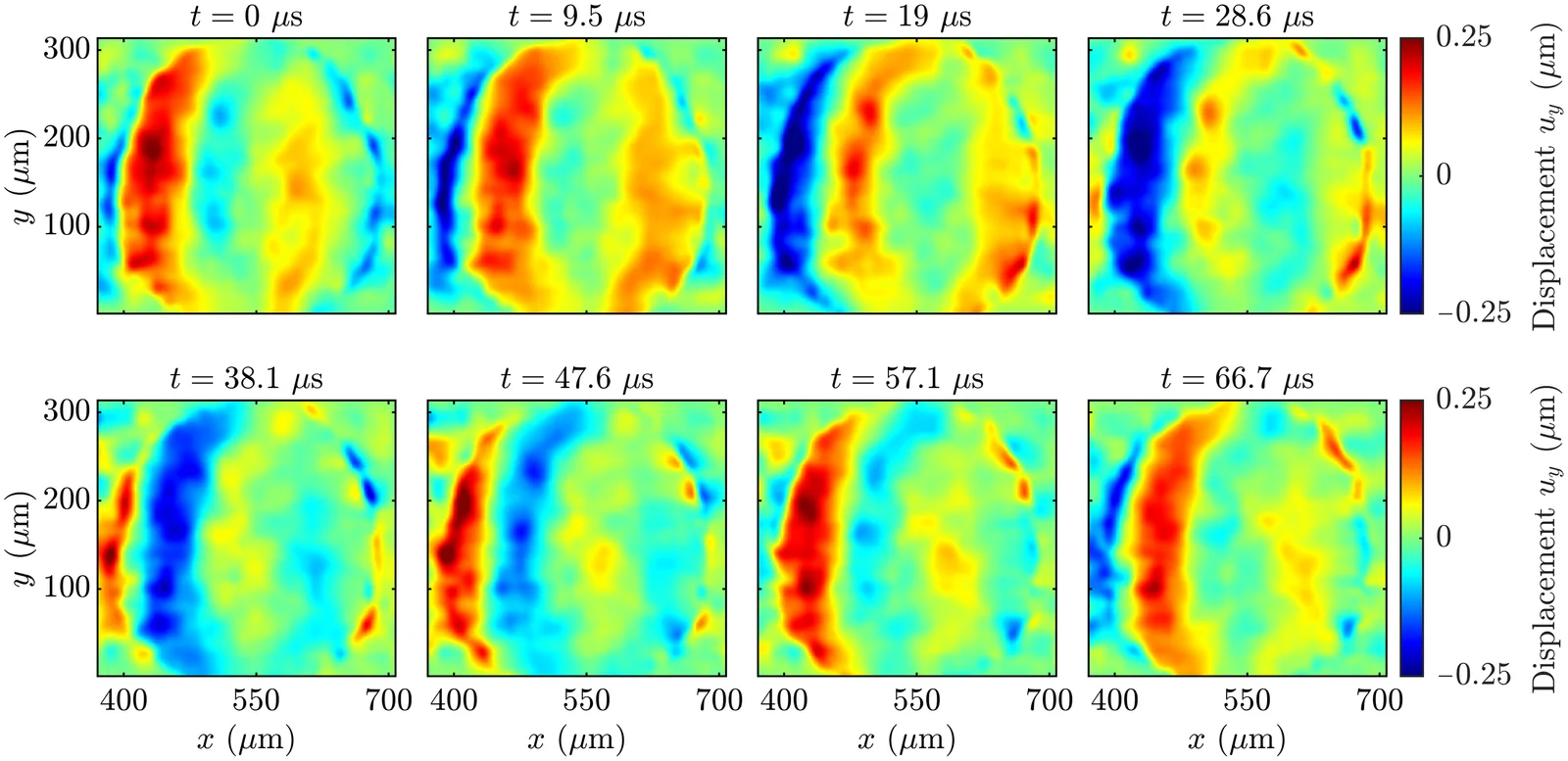
Spatiotemporal snapshots of shear-wave propagation ($u_{y}$ displacement) within a melanoma spheroid (∼277  μm diameter) embedded in a 23% polyacrylamide gel. The frames show the propagation of the shear wave through the spheroid over time, with t measured from the first frame shown and one in every two frames of the 210,000 fps recording displayed. All panels share the same color scale.

For each spheroid, the wave propagation within the ROI was analyzed with the k-space method and fitted to the Kelvin–Voigt model; for the representative spheroid shown in Fig. 9 this gave effective viscoelastic parameters of μ=0.713  kPa and η=0.033  Pa·s ($R^{2}=0.97$).

**Fig. 9:**
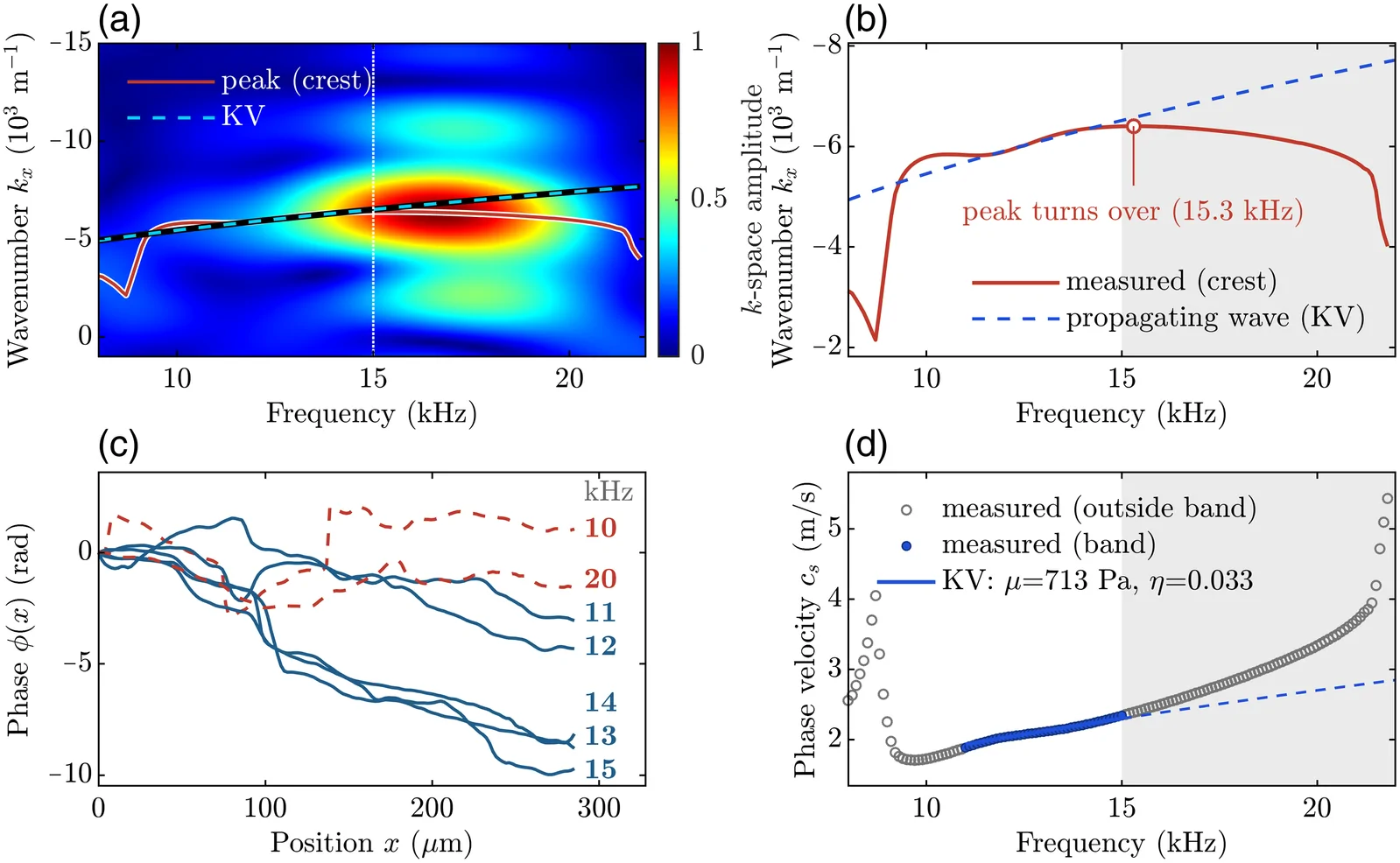
Frequency–wavenumber analysis of the shear wave in a representative melanoma spheroid. (a) Magnitude of the two-dimensional Fourier transform $U_{y}(k_{x},f)$ of the transverse displacement field, with the extracted spectral crest (solid) and the Kelvin–Voigt (KV) fit (dashed). (b) Crest wavenumber $k_{x}$ versus frequency (solid) compared with the KV dispersion (dashed); shading marks the frequencies excluded from the fit. (c) Unwrapped spatial phase profiles ϕ(x) of the transverse displacement, in-band (solid) and out-of-band (dashed). (d) Shear phase velocity $c_{s}(f)$, with in-band points (filled), out-of-band points (open), and the KV fit (solid).

Figure 9 illustrates the frequency–wavenumber analysis used to extract the shear viscoelastic parameters and, in particular, the criterion by which the fitting band is defined. The two-dimensional Fourier transform of the transverse displacement field, $U_{y}(k_{x},f)$, concentrates its energy along a single ridge [Fig. 9(a)] corresponding to the propagating shear mode. The wavenumber of this ridge, tracked as a function of frequency [Fig. 9(b)], coincides with the Kelvin–Voigt (KV) dispersion relation over an intermediate frequency range and departs from it at both ends, each departure reflecting a distinct limit of the measurement.

At the lower edge of the excitation band, the propagating ridge is no longer reliably resolved. This is directly visible in the spatial phase profiles [Fig. 9(c)]: at 10 kHz, near the tapered lower edge of the 10 to 25 kHz chirp, where the injected energy is low, the unwrapped phase ϕ(x) is essentially flat, with no appreciable gradient along the propagation direction, in contrast to the monotonic phase ramps observed within the band. The absence of a phase gradient (∂ϕ/∂x≈0), together with the loss of a well-defined crest in the f–k spectrum [Fig. 9(b)], indicates that a coherent propagating wavefront cannot be reliably extracted at this frequency; the corresponding apparent phase velocity is therefore not physically meaningful, and these frequencies are excluded from the fit. This lower limit is set by the excitation and the recovered ridge, not by the geometric resolution criterion of Sec. 2.2: at these frequencies the shear wavelength (λ≈150 to 200  μm) remains below the spheroid diameter.

At the upper edge, the crest departs from the KV dispersion in the opposite manner, turning over above 15.3 kHz. In this regime, viscous attenuation increasingly limits the distance over which the shear wave propagates within the spheroid, so that a coherent mode is no longer maintained across the field of view: the k-space crest loses definition and bends back [Fig. 9(b)], and the extracted velocity departs from the model [Fig. 9(d)]. This upper limit is consistent with the simulated amplitude decay at 23% gel concentration (Fig. 7), which indicates near-complete attenuation beyond  ≈400  μm at 20 kHz. These frequencies are likewise excluded.

Between these two limits lies the band in which the shear wave genuinely propagates: the phase advances monotonically with position, the crest follows the KV dispersion, and the phase velocity is well described by the model. The viscoelastic fit is therefore restricted to this window (12 to 15 kHz for the smaller spheroids). The same criterion, a propagating mode present and a dispersion consistent with the KV model, was used to define the fitting band of each spheroid. Because the excitation was matched to specimen size (Sec. 2.2), the larger spheroids were probed at lower frequencies and retained a well-resolved propagating mode over a correspondingly lower band (2 to 6 kHz).

Because these larger spheroids appear darker in brightfield transmission (Fig. 10), which could compromise optical-flow tracking, this criterion also served as a check on tracking quality: a monotonic phase gradient and a well-defined crest following the KV dispersion would not be recovered if the optical flow failed to track the displacement field. Both features were recovered for spheroids (c) and (d) within the 2 to 6 kHz band, and their Kelvin–Voigt fits yielded $R^{2}=0.97$ and 0.99, respectively (Fig. 10).

**Fig. 10:**
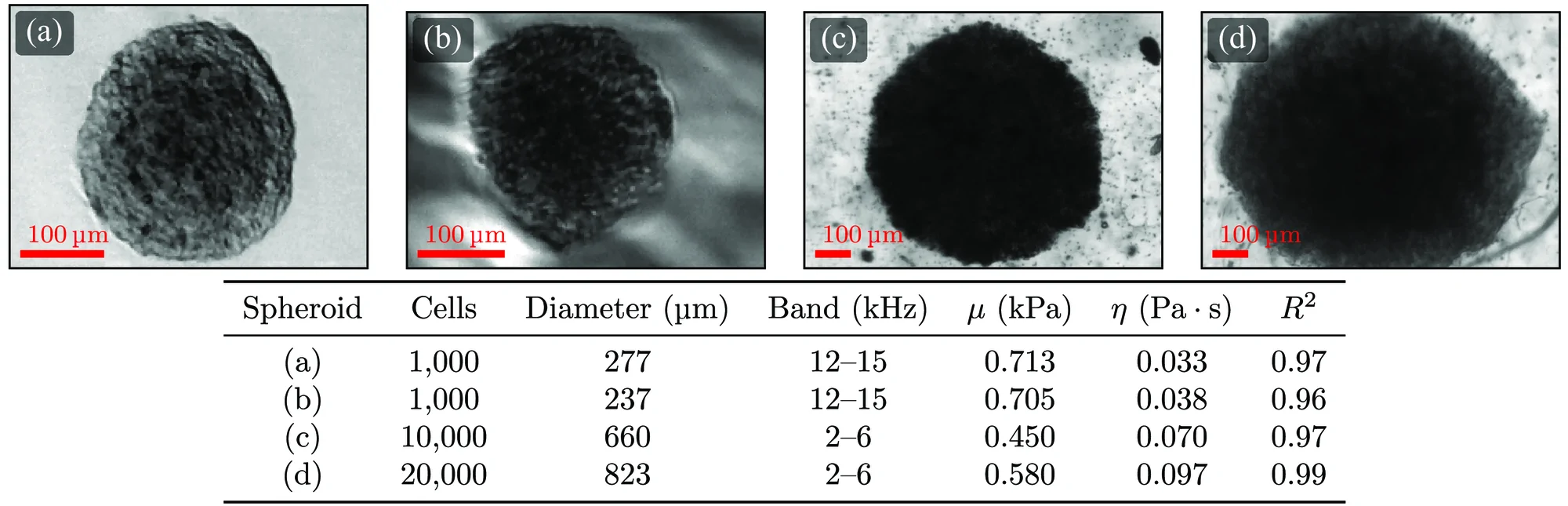
Representative spheroid images and corresponding mechanical properties for the four melanoma spheroids (a)–(d), showing cell number, diameter, analysis band, and effective Kelvin–Voigt parameters (μ, η, $R^{2}$).

The effective viscoelastic parameters obtained for the four spheroids are summarized in Fig. 10. Effective shear moduli ranged from 0.450 to 0.713 kPa and viscosities from 0.033 to 0.097  Pa·s, with $R^{2}\geq 0.96$ in all cases. Because the analysis band differs between specimens, these effective parameters are not directly comparable across spheroids and are reported as feasibility estimates rather than absolute material properties. The spheroids are also independent specimens of different sizes; the differences in μ and η between them, therefore, cannot yet be separated from the effects of size, biological variability, and fitting band.

## 4 Discussion

The proposed microelastography platform was applied to the characterization of tunable phantoms and to three-dimensional melanoma tumor spheroids. It demonstrated the technical feasibility of quantitative microscale mechanical characterization and provided estimates of effective viscoelastic parameters at kilohertz frequencies.

The following discussion situates these findings within the broader context of mechanobiology and elastography, interprets their significance, addresses methodological choices and limitations, and outlines future directions for this technology.

An important aspect of this work is the extension of optical microelastography into the multi-kilohertz near-ultrasonic regime. In shear-wave elastography, spatial resolution is fundamentally limited by the wavelength of the propagating wave. Resolving microscopic structures such as the spheroids examined here (237 to 823  μm) therefore requires shear wavelengths smaller than the feature itself.

Because the shear wavelength is inversely proportional to frequency ($\lambda =c_{s}/f$), the excitation frequency must be matched to the size of the target. For the smaller spheroids (∼237 to 277  μm), resolving wavelengths comparable to or below the feature size requires higher excitation frequencies, whereas the larger spheroids (∼660 to 823  μm) admit longer wavelengths and hence lower frequencies, which was exploited using a single-cycle 5 kHz excitation analyzed over 2 to 6 kHz.^10^

Our platform addresses this requirement through excitation adapted to specimen size, achieving minimum resolvable wavelengths of ∼110  μm in the polyacrylamide phantoms and ∼150 to 385  μm in the spheroids. This capability enables mechanical characterization at spatial scales comparable to microtissue dimensions, extending shear-wave elastography towards microscale tissue interrogation.

The experimental wavelength values (∼110 to 280  μm, Table 1) remain comparable across phantom concentrations and are consistent with the retrieved phase velocities [Fig. 6(a)]. These values are also well matched to the dimensions of the biological targets investigated here. Together, these observations indicate that the system probes mechanical behavior at multicellular spatial scales.

The phantom validation data (Table 1) also highlight a characteristic behavior of the polyacrylamide system. Increasing gel concentration from 15% to 26% produced a 27-fold increase in shear modulus (0.70 to 19.31 kPa) but only an eight-fold increase in viscosity (0.027 to 0.215  Pa·s).

This response is consistent with the underlying polymer physics. In polyacrylamide gels, the acrylamide:bis-acrylamide ratio (19:1 in this study) primarily influences viscous response, whereas crosslink density, which varies with total monomer concentration, predominantly governs elastic behavior.^44^^,^^45^ Viscosity is mainly influenced by backbone friction, whereas stiffness scales with crosslink density.^46^^,^^47^

This behavior is also reflected in the coefficients of variation observed across independent replicates (Table 1), which are generally smaller for viscosity (${\mathrm{CV}}_{\eta }=1.2\%$ to 20.3%) than for shear modulus (${\mathrm{CV}}_{\mu }=7.1\%$ to 34.5%).

Application of our system to the four melanoma spheroids yielded effective shear moduli of μ≈0.45 to 0.71 kPa and effective viscosities of η≈0.03 to 0.10 Pa⋅s within their respective interrogation bands. These values provide quantitative estimates of viscoelastic parameters for tumor spheroid models within a frequency range that remains relatively underexplored.

The measured shear modulus is broadly consistent with reported values for other tumor spheroids obtained using techniques such as atomic force microscopy (AFM) and magnetic microelastography, which typically fall between a few hundred pascals and several kilopascals.^48^^–^^51^ However, it remains approximately an order of magnitude lower than stiffness values reported for excised human melanoma tissue (∼25.6  kPa).^52^

This discrepancy likely reflects, at least in part, biological differences between tumor spheroids and mature tumors *in vivo* rather than experimental artifacts. Several factors may contribute to the higher stiffness reported for *in vivo* tumors. First, mature tumors possess a dense, highly cross-linked and remodelled extracellular matrix (ECM), a process driven by cancer-associated fibroblasts, which are absent from our monoculture system.^53^^,^^54^ Second, tumors growing within confined physiological environments experience substantial solid compressive stress, which can increase their apparent stiffness.^55^^,^^56^

Importantly, tumor mechanics encompass more than stiffness alone; viscoelastic properties also influence cancer progression and treatment response.^3^ By isolating cancer cells from their stromal surroundings, our system primarily probes the intrinsic mechanical behavior of the cancer cell collective.^57^

Furthermore, whilst our analysis yields a bulk effective value for each spheroid, the spatiotemporal displacement data (Fig. 8) provide qualitative indications of spatial variation in wave propagation. Qualitative visual inspection suggests that shear waves propagate slightly faster and attenuate more strongly near the spheroid periphery than in the core.

This qualitative pattern is compatible with peripheral stiffening mechanisms reported in tumor spheroids, including increased cell density, enhanced cell–cell adhesion, and nascent extracellular matrix deposition.^27^^,^^58^^,^^59^ However, wave scattering and diffraction at the curved gel–spheroid interface may also contribute to the observed spatial variation in apparent wave velocity. The interaction of a quasi-planar shear wave with a spherical inclusion of markedly different mechanical impedance can produce spatially heterogeneous displacement fields even in mechanically homogeneous targets.^35^

Distinguishing between true mechanical heterogeneity and wave-propagation artifacts would require spatially resolved inversion methods or numerical modeling of the full wave-scattering problem, which represents an important direction for future work.

### 4.1 Limitations and Future Directions

A common methodological limitation in elastography studies is the use of the two-parameter Kelvin–Voigt model, which simplifies the complex rheology of biological tissues that often exhibit power-law or fractional viscoelastic behavior.^10^^,^^46^^,^^47^ In the present study, fractional Kelvin–Voigt fits yielded exponents consistently close to unity (α≈1 across phantoms), indicating that within the 5 to 25 kHz bandwidth the classical formulation provides a reasonable approximation of the observed dispersion behavior.^41^^,^^42^ Nevertheless, broader-bandwidth studies may benefit from fractional-order models.

The analysis further assumes locally planar wavefronts within the equatorial region of interest, rather than planarity of the construct itself, the same assumption routinely made for the cylindrical wavefronts of acoustic radiation force excitation, treated as locally planar sufficiently far from the source. The ROI selection minimizes the influence of boundary conditions, diffraction, and optical distortions, ensuring that the measured response primarily reflects the intrinsic mechanical behavior of the spheroid. It is adequate for the approximately ellipsoidal geometry typical of tumor spheroids, but markedly irregular constructs would require modeling of the full wave-scattering problem or locally resolved inversion.

In addition, although spatially resolved approaches such as local phase-gradient methods,^43^^,^^60^ pixel-wise reconstruction,^61^^,^^62^ or noise-correlation techniques^63^^,^^64^ provide powerful strategies for mapping mechanical heterogeneity, the spatially averaged methodology adopted here is appropriate for the platform-validation and effective-property objectives of this study. Future work should implement these higher-resolution approaches to quantify fully the mechanical gradients qualitatively observed in the spheroid measurements. In the present spheroid experiments, the limited propagation distance at the upper frequencies and the small number of wavelengths across the construct favor robust ROI-averaged estimation over local pixel-wise inversion.

A further limitation concerns the mechanical impedance mismatch between the embedding gel (μ≈10.14  kPa) and the spheroids (μ≈0.45 to 0.71 kPa). This contrast may introduce residual wave scattering and partial reflection at the gel–spheroid interface, potentially influencing the estimated viscoelastic parameters. Although directional filtering suppresses reflected wave components^39^ and the equatorial ROI selection minimizes curvature-induced diffraction effects, interface-mediated wave distortion cannot be completely excluded. Future work should investigate impedance-matched embedding media or wave-scattering correction models to better isolate the intrinsic mechanical response of the spheroid.

All measurements were conducted at ambient temperature (22°C to 24°C) without active thermal control. Owing to the thermo-viscoelastic nature of soft tissues, this may introduce a systematic bias whose magnitude and direction depend on the specific material properties.^65^^,^^66^ Although some tissues stiffen at physiological temperature, others such as brain tissue soften markedly, meaning that ambient measurements can either under- or overestimate *in vivo* properties.^67^^,^^68^ This sensitivity is particularly pronounced in the acrylamide-based phantoms used here. Incorporating a temperature-controlled stage to maintain samples at 37°C, therefore, represents an important step toward improved physiological relevance.

The biological scope of this study is deliberately limited. Measurements were performed on four melanoma spheroids of a single cell line and different sizes, constituting a proof-of-concept demonstration of technical feasibility rather than a statistically powered biological characterization. Consequently, the reported viscoelastic parameters should be interpreted as indicative estimates rather than representative values for melanoma spheroid mechanics. Robust biological conclusions will require repeatability measurements on the same specimen and measurements across additional cell lines and replicates, including co-culture models incorporating cancer-associated fibroblasts and stromal cells to better recapitulate the tumor microenvironment.^69^

Finally, the present approach is restricted to optically transparent or translucent samples. Nevertheless, the measurements show that melanoma spheroids provide sufficient endogenous optical contrast for displacement tracking. To extend applicability to opaque ex vivo tissue specimens, future work will explore integrating the high-frequency actuation and analysis framework with imaging modalities offering greater penetration depth, such as optical coherence tomography.^70^

## 5 Conclusions

This work demonstrates kilohertz-range viscoelastic characterization of three-dimensional biological constructs at sub-millimeter resolution. The optical microelastography platform combines quasi-planar shear-wave excitation using a piezoelectric actuator, high-speed imaging (up to 210,000 fps), and k-space dispersion analysis to estimate frequency-dependent viscoelastic parameters through a Kelvin–Voigt model.

Validation across polyacrylamide phantoms spanning 0.70 to 19.31 kPa over the 5 to 25 kHz frequency range demonstrated robust performance, with experimental phase-velocity dispersion showing excellent agreement with finite-element predictions (RMSE<0.16  m/s, mean relative error <3%, $R^{2}>0.96$). The minimum resolvable shear wavelength of ∼110  μm in the phantoms confirms effective mechanical interrogation at multicellular length scales.

Application to four melanoma tumor spheroids (237 to 823 µm diameter) yielded effective viscoelastic parameters of μ=0.450 to 0.713 kPa and η=0.033 to 0.097  Pa·s ($R^{2}\geq 0.96$) within specimen-specific analysis bands, demonstrating feasibility in biologically relevant three-dimensional constructs. Future studies with larger cohorts and multiple cell lines will establish the statistical robustness required for biological inference.

This quasi-planar excitation approach reduces geometric spreading artifacts associated with traditional point-source methods and provides a noninvasive optical tool for studying frequency-dependent mechanical behavior in mechanobiology and tissue engineering. This platform provides a foundation for quantitative viscoelastic characterization of engineered tissue constructs and three-dimensional biological models at kilohertz frequencies.

## Data Availability

The datasets generated and analyzed during this study, together with the MATLAB processing scripts used for the k-space dispersion analysis, are available from the corresponding author upon reasonable request.
